# Liver-First Approach for Synchronous Colorectal Metastases: Analysis of 7360 Patients from the LiverMetSurvey Registry

**DOI:** 10.1245/s10434-021-10220-w

**Published:** 2021-07-01

**Authors:** Felice Giuliante, Luca Viganò, Agostino M. De Rose, Darius F. Mirza, Réal Lapointe, Gernot Kaiser, Eduardo Barroso, Alessandro Ferrero, Helena Isoniemi, Santiago Lopez-Ben, Irinel Popescu, Jean-Francois Ouellet, Catherine Hubert, Jean-Marc Regimbeau, Jen-Kou Lin, Oleg G. Skipenko, Francesco Ardito, René Adam

**Affiliations:** 1 Hepatobiliary Surgery Unit, Foundation “Policlinico Universitario A. Gemelli”, IRCCS, Catholic University, Rome, Italy; 2 Division of Hepatobiliary and General Surgery, Department of Surgery, IRCCS – Humanitas Clinical and Research Center, Rozzano, Milan, Italy; 3 HPB Surgery, University Hospitals Birmingham NHS Foundation Trust, Birmingham, UK; 4 Hepatopancreatobiliary Surgery and Liver Transplantation Service, Centre Hospitalier de l’Université de Montréal (CHUM), Montreal, QC Canada; 5 Department of General, Visceral and Transplantation Surgery, University Hospital of Essen, Essen, Germany; 6 HBP and Transplantation Centre, Curry Cabral Hospital, Lisbon Central Hospitals Centre, Lisbon, Portugal; 7 Department of General and Oncological Surgery, “Umberto I” Mauriziano Hospital, Turin, Italy; 8 Department of Liver Surgery and Transplantation, Helsinki University, Helsinki, Finland; 9 Hepatobiliary and Pancreatic Surgery Unit, Department of Surgery, Dr. Josep Trueta Hospital, IdlBGi, Girona, Spain; 10 Department of Surgery and Transplantation, Fundeni Clinical Institute, Bucharest, Romania; 11 CHU de Québec – Université Laval, Quebec City, QC Canada; 12 Department of HBP Surgery, Cliniques Universitaires Saint-Luc, Université Catholique de Louvain, Leuven, Belgium; 13 Department of Oncology and Digestive Surgery, CHU Amiens-Picardie, Amiens, France; 14 Division of Colon and Rectal Surgery, Department of Surgery, Taipei Veterans General Hospital and School of Medicine, National Yang-Ming University, Taipei, Taiwan; 15 Research Center of Surgery, Russian Academy of Medical Science, Moscow, Russia; 16 Department of Surgery, Paul-Brousse Hospital, Assistance Publique Hôpitaux de Paris, Centre Hépato-Biliaire, Villejuif, France

## Abstract

**Background:**

The liver-first approach in patients with synchronous colorectal liver metastases (CRLM) has gained wide consensus but its role is still to be clarified. We aimed to elucidate the outcome of the liver-first approach and to identify patients who benefit at most from this approach.

**Methods:**

Patients with synchronous CRLM included in the LiverMetSurvey registry between 2000 and 2017 were considered. Three strategies were analyzed, i.e. liver-first approach, colorectal resection followed by liver resection (primary-first), and simultaneous resection, and three groups of patients were analyzed, i.e. solitary metastasis, multiple unilobar CRLM, and multiple bilobar CRLM. In each group, patients from the three strategy groups were matched by propensity score analysis.

**Results:**

Overall, 7360 patients were analyzed: 4415 primary-first, 552 liver-first, and 2393 simultaneous resections. Compared with the other groups, the liver-first group had more rectal tumors (58.0% vs. 31.2%) and higher hepatic tumor burden (more than three CRLMs: 34.8% vs. 24.0%; size > 50 mm: 35.6% vs. 22.8%; *p <* 0.001). In patients with solitary and multiple unilobar CRLM, survival was similar regardless of treatment strategy, whereas in patients with multiple bilobar metastases, the liver-first approach was an independent positive prognostic factor, both in unmatched patients (3-year survival 65.9% vs. primary-first 60.4%: hazard ratio [HR] 1.321, *p =* 0.031; vs. simultaneous resections 54.4%: HR 1.624, *p <* 0.001) and after propensity score matching (vs. primary-first: HR 1.667, *p =* 0.017; vs. simultaneous resections: HR 2.278, *p =* 0.003).

**Conclusion:**

In patients with synchronous CRLM, the surgical strategy should be decided according to the hepatic tumor burden. In the presence of multiple bilobar CRLM, the liver-first approach is associated with longer survival than the alternative approaches and should be evaluated as standard.

**Supplementary Information:**

The online version contains supplementary material available at 10.1245/s10434-021-10220-w.

Colorectal cancer is the second most common cancer in Europe and the second cause of cancer-related mortality worldwide.^1^,^2^ At first diagnosis, up to 20% of patients with colorectal cancer have liver metastases (CRLM) that reflect poor tumor biology and prognosis.^3^–^5^ Nevertheless, complete resection of both primary tumor and CRLM improves survival expectancy compared with systemic therapies.^6^–^10^

The management of patients with synchronous CRLM requires complex multidisciplinary evaluation as long as colorectal surgery, hepatic surgery, and chemotherapy are combined with appropriate timing. Traditional approaches schedule either simultaneous colorectal and hepatic resection or colorectal resection followed by liver resection (primary tumor-first approach), administering chemotherapy in the perioperative period. In 2006, Mentha et al. proposed a reverse strategy (liver-first approach), scheduling first liver resection and then primary tumor resection to prioritize the removal of the most prognostically relevant disease (liver metastases) and to ease the inclusion of radiotherapy for locally advanced rectal tumors.^11^ Even if appealing, the liver-first approach failed to demonstrate its superiority over the alternatives, mainly because of different patient selection in a determined single institution.^12^–^22^ To date, the choice of treatment strategy of synchronous CRLM relies on a case-by-case evaluation by every single multidisciplinary expert team rather than on robust evidence or proven benefits.

The present study aims to elucidate the role of reverse strategy in the management of patients with synchronous CRLM, analyzing the impact of treatment strategy in patients at different tumor burden. Thanks to the analysis of a large multicenter international cohort of patients, we have solid expectancy to identify patients who benefit most from a reverse strategy.

## Methods

All patients with synchronous CRLM included in the LiverMetSurvey registry (www. livermetsurvey-arcad.org) undergoing liver surgery between January 2000 and December 2017 were reviewed. The characteristics of the LiverMetSurvey registry have been previously reported.^23^–^25^ Patients were divided into three groups according to the adopted surgical strategy: (1) colorectal resection followed by liver resection (classical approach, primary-first group); (2) liver resection followed by colorectal resection (reverse strategy, liver-first group); (3) simultaneous colorectal and hepatic resection (simultaneous group). Exclusion criteria were unresected patients, missing or incomplete data regarding surgical procedures, delay between primary tumor and liver resection of > 1 year in the primary-first group, and two-stage hepatectomy.

### Study Design

The liver-first group was compared with the other two approaches in terms of short- and long-term outcomes. Furthermore, we compared the outcome of the three approaches in three subgroups of patients, classified according to their hepatic tumor burden: patients with solitary metastasis, patients with multiple unilobar metastases, and patients with multiple bilobar metastases. Considering short-term outcome analysis, in the primary-first and liver-first groups we considered only complications related to liver resection because complications related to colorectal resection are not reported in the registry. We analyzed 90-day mortality, overall morbidity, hepatic complication (i.e. liver failure, bile leak, infected perihepatic collection, bleeding from the cut surface, or ascites), and infectious complication rates. Considering survival analysis, in the three subgroups (solitary, multiple unilobar, and multiple bilobar CRLM) we compared the three approaches before and after propensity score matching of patients (see details in the Statistical Analysis section). Of note, the registry includes only patients operated on for CRLM. Consequently, even if all data regarding patients’ history are collected, LiverMetSurvey data do not allow an intention-to-treat analysis to be performed of patients with synchronous CRLM.

### Statistical Analysis

Categorical variables were compared using the Chi-square test or Fisher’s exact test, as appropriate, whereas continuous variables were compared using parametric (unpaired *t* test) or non-parametric (Mann–Whitney *U-*test) tests, as appropriate. The Kaplan–Meier method was used to estimate survival probabilities, which were compared using the log-rank test. Overall survival (OS) and recurrence-free survival (RFS) were calculated from the date of liver surgery. Furthermore, we calculated OS from the date of diagnosis of CRLM, and OS from the date of completion of surgical strategy, i.e. from the date of liver surgery in the primary-first and simultaneous groups and from the date of colorectal surgery in the liver-first group. Cox proportional hazards regression models were used to evaluate the association of the relevant clinicopathological factors with prognosis. Variables with a *p* value < 0.10 at univariate analysis and those associated with outcome in the literature were included in the multivariable analysis. Carcinoembryonic antigen (CEA) values and RAS status were not included in the analysis because the former was missing in a high proportion of patients (34%) and the latter was not reported in the registry.

When we performed the survival analysis of the three subgroups of patients (solitary CRLM, multiple unilobar CRLM, and multiple bilobar CRLM), we used a propensity score analysis to overcome biases owing to the different distribution of covariates among patients in the three approach groups. In every subgroup, two separate propensity score analyses were performed: the first to match (1:2) patients in the liver-first group with patients in the primary-first group; and the second to match (1:1) patients in the liver-first group with patients in the simultaneous group. Separate multivariable logistic regression models were performed (one per each propensity score analysis) to predict the probability of each patient being submitted to a surgical approach on the basis of the following covariates: age, year of resection, primary tumor site, N status of the primary tumor, number of CRLMs, size of CRLM, extrahepatic disease, and preoperative chemotherapy. The nearest-neighbor matching method was used. Matching to 5 decimal points was performed, followed by 4-, 3-, and 2-point matching. Cases whose propensity score deviated > 0.01 were considered unmatched and were hence excluded.

All analyses were carried out using SPSS version 25 (IBM Corporation, Armonk, NY, USA) and STATA version 14 (StataCorp LLC, Cary, NC, USA).

## Results

Overall, 12,744 patients with synchronous CRLM who were included in the LiverMetSurvey registry were considered. A total of 5384 patients were excluded (908 unresected patients, 2835 patients with missing or incomplete data regarding surgical procedures, 909 patients with a delay between primary tumor and liver resection > 1 year, and 732 two-stage hepatectomies), resulting in 7360 patients being analyzed: 4415 patients (60.0%) in the primary-first group, 552 (7.5%) patients in the liver-first group, and 2393 (32.5%) patients in the simultaneous group (electronic supplementary Fig. 1).

The liver-first approach increased over time, from 2.4% before 2007 to 5.9% in the period 2007–2011, and to 12.6% later on (*p <* 0.001), while the primary-first approach decreased (68.3%, 58.2%, and 57.3%, respectively; *p <* 0.001) and the simultaneous group remained stable  (≈ 30%). During the study period, the number of resected metastases did not have a significant increase (three or more nodules: 23.9% before 2007, 24.8% in the period 2007–2011, and 26.1% after 2011), while metastasis size decreased (>50 mm: 27.8%, 23.1, and 22.2%, respectively; *p =* 0.001). The proportion of patients undergoing preoperative chemotherapy progressively increased (42.1%, 50.9%, and 54.1%, respectively; *p* < 0.001).

The three groups of patients had major differences. The liver-first group included more patients with rectal tumors (58.0% vs. 31.2% in the other groups; *p <* 0.001) and fewer patients with right/transverse colon cancers (12.4% vs. 26.4%; *p <* 0.001). Among patients with rectal tumors, patients undergoing a reverse strategy more often had pelvic radiotherapy (56.6% vs. 33.3%; *p <* 0.001). The liver-first group had the highest tumor burden in terms of CRLM number (more than three in 34.8% vs. 24.0%; *p <* 0.001), size (>50 mm in 35.6% vs. 22.8%; *p <* 0.001), and bilobar distribution (51.5% vs. 38.8%, *p <* 0.001). In comparison with the primary-first strategy, the reverse strategy was not only associated with more preoperative chemotherapy (75.7% vs. 57.0%; *p <* 0.001) but also with a shorter treatment (more than six cycles 57.2% vs. 62.78%; *p =* 0.044) and a shorter interval between the two surgical procedures (colorectal and liver resection, mean 92 vs. 162 days; *p* < 0.001). Table 1 summarizes the patient characteristics.

**Table 1:** Patient characteristics according to the surgical strategy

Variable	Liver-first (*n* = 552)	Primary-first (*n* = 4415)	*p* value Primary-first versus liver-first	Simultaneous (*n* = 2393)	*p* value Simultaneous versus liver-first
Sex (female)	205 (37.1)	1794 (40.6)	0.114	985 (41.2)	0.082
Age > 70 years	111 (20.1)	896 (20.3)	0.918	658 (27.5)	< 0.001
*Primary cancer*					
Tumor site					
Right/transverse colon	68/547 (12.4)	993/4318 (23.0)	< 0.001	778/2383 (32.7)	< 0.001
Left colon	162/547 (29.6)	1994/4318 (46.2)		848/2383 (35.6)	
Rectum	317/547 (58.0)	1331/4318 (30.8)		757/2383 (31.8)	
T stage (T3-4)	416/427 (97.4)	3795/3834 (99.0)	0.005	2078/2106 (98.7)	0.056
LN metastases (N1)	325/499 (65.1)	2971/4080 (72.8)	< 0.001	1553/2232 (69.6)	0.053
Radiotherapy if rectal cancer	172/304 (56.6)	389/1250 (31.1)	< 0.001	274/739 (37.1)	< 0.001
*Liver metastases*					
Single metastasis	144/532 (27.1)	1501/4198 (35.8)	< 0.001	1118/2308 (48.4)	< 0.001
Metastases > 3	185/532 (34.8)	1129/4198 (26.9)	< 0.001	430/2308 (18.6)	< 0.001
Metastasis > 5 cm	173/486 (35.6)	881/3827 (23.0)	< 0.001	462/2069 (22.3)	< 0.001
Bilateral metastases	281/546 (51.5)	1837/4345 (42.3)	< 0.001	762/2354 (32.4)	< 0.001
R1 resection^a^	97/463 (21)	592/3585 (16.5)	0.017	193/1715 (11.3)	< 0.001
Extrahepatic disease	35 (6.3)	187 (4.2)	0.024	160 (6.7)	0.768
Lung metastases	28 (5.1)	123 (2.8)	0.003	73 (3.1)	0.019
LN metastases	3 (0.5)	20 (0.5)	0.768	23 (1.0)	0.344
Hepatic pedicle LNs	–	6	1.000	6	0.601
CEA > 200 ng/mL	19/383 (5.0)	128/2912 (4.4)	0.614	104/1568 (6.6)	0.227
*Chemotherapy data*					
Neoadjuvant chemotherapy	418/552 (75.7)	2516/4413 (57.0)	< 0.001	757/2393 (31.6)	< 0.001
Chemotherapy lines > 1	52/418 (12.4)	264/2516 (10.5)	0.234	104/755 (13.8)	0.519
Chemotherapy cycles > 6	210/367 (57.2)	1380/2200 (62.7)	0.044	369/647 (57.0)	0.954
Response to chemotherapy					
CR	10/388 (2.6)	72/2268 (3.2)	0.004	26/704 (3.7)	0.382
PR	305/388 (78.6)	1578/2268 (69.6)		522/704 (74.2)	
SD	60/388 (15.5)	500/2268 (22.1)		125/704 (17.8)	
PD	13/388 (3.4)	118/2268 (5.2)		31/704 (4.4)	
*Resection details*					
Type of resection					
Anatomic	215/541 (39.7)	1806/4281 (42.2)	0.008	720/2308 (31.2)	< 0.001
Anatomic + non-anatomic	183/541 (33.8)	1180/4281 (27.6)		345/2308 (15.0)	
Non-anatomic	143/541 (26.4)	1295/4281 (30.3)		1243/2308 (53.9)	
Major hepatectomy	145/357 (40.6)	1219/3202 (38.1)	0.348	290/1973 (14.7)	< 0.001
Associated intraoperative thermal ablation	77/552 (14.0)	524/4413 (11.9)	0.159	165/2392 (6.9)	< 0.001

Data are expressed as *n/N* (%) unless otherwise specified

*LN* lymph node, *CEA* carcinoembryonic antigen, *CR* complete response, *PR* partial response, *SD* stable disease, *PD* progression of disease

^a^R1 resection refers to the surgical margin of liver resection

In the whole series, the liver-first group had 90-day mortality, overall morbidity, and hepatic morbidity rates similar to the other groups. Staged resections (liver-first and primary-first groups) had a lower infectious complication rate than the simultaneous group (11.4% vs. 18.6%; *p* < 0.001). The same results were observed considering patients with solitary or multiple unilobar metastases. Considering patients with multiple bilobar metastases, the liver-first group had 90-day mortality, overall morbidity, and infectious morbidity rates similar to the primary-first group (2.3% vs. 2.0%, 31.1% vs. 30.4%, and 12.0% vs. 11.8%, respectively), but lower than the simultaneous group (vs. 5.1%, *p =* 0.052; vs. 39.9%, *p =* 0.016; vs. 20.1%, *p =* 0.006, respectively). Hepatic complication rates were similar among the three groups. Considering patients who require a major hepatectomy, independently of the tumor burden, mortality was lower in the liver-first group (4.8%) and primary-first group (2.9%) than in the simultaneous group (8.1%; *p* < 0.001). Short-term outcomes are detailed in electronic supplementary Table 1.

### Survival Analysis

#### Whole Series

After a median follow-up of 37 months, 5-year OS after liver resection was 46.6% (median OS 53.0 months, median OS since diagnosis of CRLM 59.3 months). The liver-first approach had the longest OS (at 5 years 51.4%; median OS 65.4 months), followed by the primary-first approach (47.1%, 53.9 months; *p =* 0.213) and the simultaneous group (44.8%, 51.0 months, *p =* 0.014 vs. the liver-first group) (electronic supplementary Fig. 2). Median OS since diagnosis of CRLM was 72.4, 61.0, and 53.3 months in the liver-first, primary-first, and simultaneous groups, respectively. Considering RFS, recurrence data were available for 7084 patients. The liver-first group had the lowest recurrence rate (36.8% [200/544] vs. 38.7% [890/2302] in the simultaneous group, and 45.6% [1931/4238] in the colon-first group; *p <* 0.001). The liver-first group had RFS similar to the simultaneous group (liver-first group: 5-year RFS 37.5% and median RFS 28.2 months; simultaneous group: 5-year RFS 37.9% and median RFS 30.5 months), higher than the primary-first group (5-year RFS 34.6% and median RFS 23.9 months, *p =* 0.043).

At multivariable analysis, OS after liver resection of the liver-first group was better than that of the simultaneous group (hazard ratio [HR] 1.298 for the latter group; *p =* 0.036), and similar to that of the primary-first group (HR 1.212; *p* = 0.100) (electronic supplementary Table 2).

#### Solitary Metastasis

**Fig. 1:**
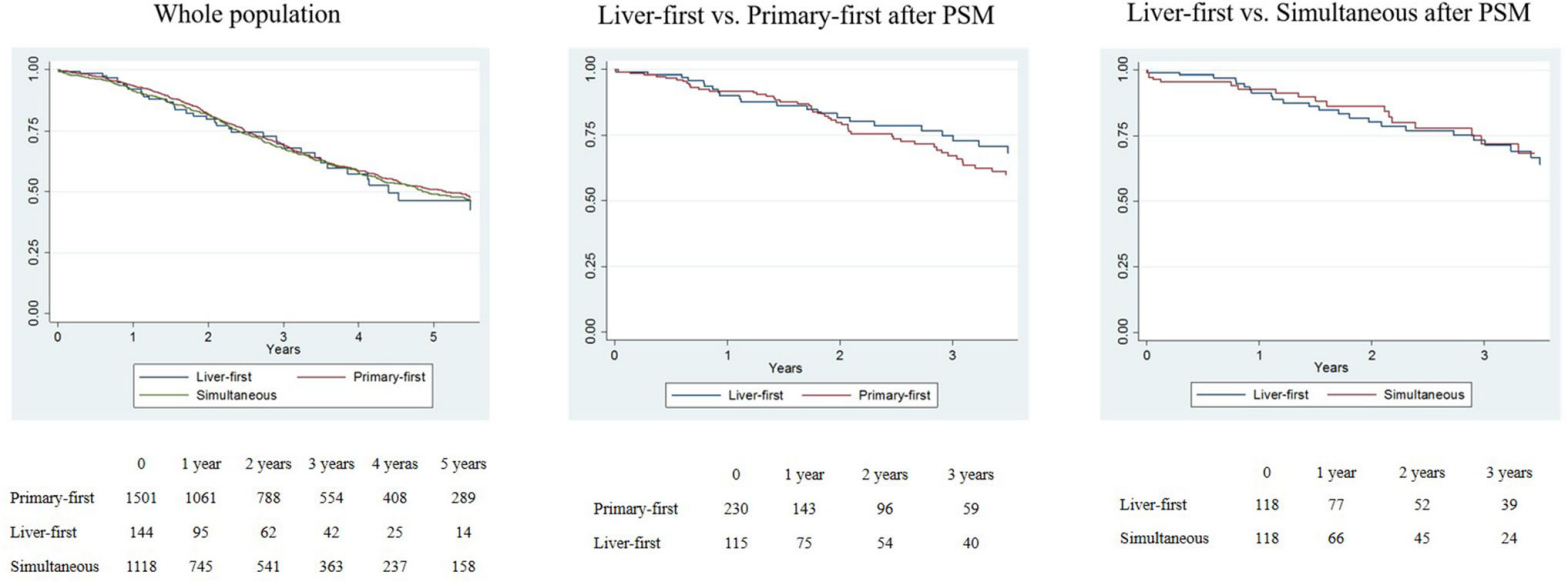
Overall survival in patients with solitary metastasis according to the treatment strategy. **a** Whole population, **b** liver-first group versus primary-first group after PSM, **c** liver-first group versus simultaneous group after PSM. *PSM* propensity score matching

In patients with solitary metastasis, OS was not associated with treatment strategy (liver-first group vs. primary-first group: 3-year OS 69.6% vs. 69.1%, HR 0.932, *p =* 0.672; and liver-first group vs. simultaneous group: 3-year OS 69.6% vs. 67.8%, HR 0.979, *p* = 0.898) (Fig. 1a). After propensity score matching, we compared (1) 115 patients in the liver-first group versus 230 patients in the primary-first group; and (2) 118 patients in the liver-first group versus 118 patients in the simultaneous group. The characteristics of the groups were similar after matching (Table 2). The liver-first group had survival similar to both the primary-first group (3-year OS 74.9% vs. 67.1%, HR 0.842, *p =* 0.445) and the simultaneous group (73.3% vs. 71.9%, HR 1.218, *p* = 0.486) (Fig. 1b, c). Multivariable analyses in matched groups confirmed no association between treatment strategy and OS after liver resection. Similarly, there was no association between treatment strategy and OS since CRLM diagnosis, OS after completion of treatment strategy, and RFS (data not shown).

**Table 2:** Patient characteristics after propensity score matching

	Liver-first	Primary-first	*p* value Liver-first versus primary-first	Liver-first	Simultaneous	*p* value Liver-first versus simultaneous
*Single metastasis*						
	n = 115	n = 230		n = 118	n = 118	
Age > 70 years	25 (21.7)	44 (19.1)	0.568	29 (24.6)	30 (25.4)	0.881
Year of surgery						
2000–2006	10 (8.7)	19 (8.3)	0.990	10 (8.5)	10 (8.5)	0.990
2007–2011	37 (32.2)	74 (32.2)		37 (31.4)	36 (30.5)	
2012–2017	68 (59.1)	137 (59.6)		71 (60.2)	72 (61.0)	
Tumor site						
Right/transverse colon	16 (13.9)	31 (13.5)	0.992	16 (13.6)	16 (13.6)	0.902
Left colon	34 (29.6)	69 (30.0)		33 (28.0)	30 (25.4)	
Rectum	65 (56.5)	130 (56.5)		69 (58.5)	72 (61.0)	
N+ primary tumor	76 (66.1)	153 (66.5)	0.936	78 (66.1)	80 (67.8)	0.782
Metastases diameter > 50 mm	38 (33.0)	74 (32.2)	0.871	39 (33.1)	36 (30.5)	0.675
Extrahepatic disease	2 (1.7)	4 (1.7)	1.000	2 (1.7)	2 (1.7)	1.000
Preoperative chemotherapy	69 (60.0)	135 (58.7)	0.816	65 (55.1)	64 (54.2)	0.896
*Unilobar multiple metastases*						
	n = 84	n = 168		n = 73	n = 73	
Age > 70 years	14 (16.7)	19 (11.3)	0.235	13 (17.8)	10 (13.7)	0.496
Year of surgery						
2000–2006	4 (4.8)	9 (5.4)	0.952	4 (5.5)	5 (6.8)	0.862
2007–2011	34 (40.5)	65 (38.7)		31 (42.5)	33 (45.2)	
2012–2017	46 (54.8)	94 (56.0)		38 (52.1)	35 (47.9)	
Tumor site						
Right/transverse colon	13 (15.5)	28 (16.7)	0.935	13 (17.8)	11 (15.1)	0.827
Left colon	25 (29.8)	52 (31.0)		21 (28.8)	24 (32.9)	
Rectum	46 (54.8)	88 (52.4)		39 (53.4)	38 (52.1)	
N+ primary tumor	53 (63.1)	104 (61.9)	0.854	45 (61.6)	46 (63.0)	0.864
Number of metastases > 3	15 (17.9)	27 (16.1)	0.720	15 (20.5)	15 (20.5)	1.000
Number of metastases [mean (range)]	2.8 (2–9)	2.7 (2–7)	0.297	2.9 (2–9)	2.9 (2–8)	0.952
Metastases diameter > 50 mm	22 (26.2)	45 (26.8)	0.920	18 (24.7)	21 (28.8)	0.575
Extrahepatic disease	–	–	1.000	1 (1.4)	1 (1.4)	1.000
Preoperative chemotherapy	60 (71.4)	120 (71.4)	1.000	49 (67.1)	50 (68.5)	0.859
*Bilobar multiple metastases*						
	n = 163	n = 326		n = 135	n = 135	
Age > 70 years	33 (20.2)	66 (20.2)	1.000	25 (18.5)	23 (17.0)	0.750
Year of surgery						
2000–2006	7 (4.3)	11 (3.4)	0.737	7 (5.2)	7 (5.2)	1.000
2007–2011	57 (35.0)	124 (38.0)		55 (40.7)	55 (40.7)	
2012–2017	99 (60.7)	191 (58.6)		73 (54.1)	73 (54.1)	
Tumor site						
Right/transverse colon	25 (15.3)	46 (14.1)	0.924	23 (17.0)	24 (17.8)	0.984
Left colon	58 (35.6)	120 (36.8)		45 (33.3)	44 (32.6)	
Rectum	80 (49.1)	160 (49.1)		67 (49.6)	67 (49.6)	
N+ primary tumor	113 (66.0)	215 (69.3)	0.454	92 (68.1)	93 (68.9)	0.896
Number of metastases > 3	96 (58.9)	192 (58.9)	1.000	76 (56.3)	77 (57.0)	0.902
Number of metastases [mean (range)]	5.3 (2–25)	5.0 (2–36)	0.309	5.2 (2–25)	4.6 (2–20)	0.217
Metastases diameter > 50 mm	51 (31.3)	109 (33.4)	0.633	46 (34.1)	44 (32.6)	0.796
Extrahepatic disease	2 (1.2)	4 (1.2)	1.000	2 (1.5)	2 (1.5)	1.000
Preoperative chemotherapy	141 (86.5)	275 (84.4)	0.530	113 (83.7)	114 (84.4)	0.868

#### Multiple Unilobar Metastases

**Fig. 2:**
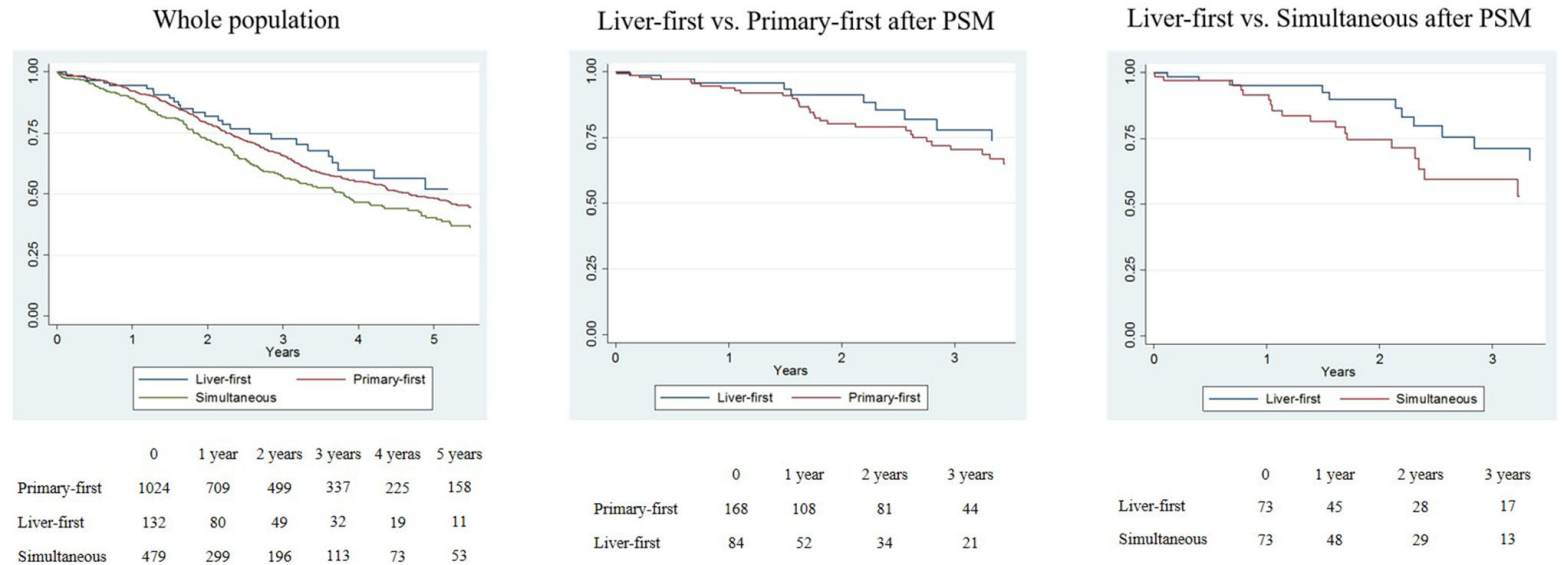
Overall survival in patients with multiple unilobar metastases according to the treatment strategy. **a** Whole population, **b** liver-first group versus primary-first group after PSM, **c** liver-first group versus simultaneous group after PSM. *PSM* propensity score matching

In patients with multiple unilobar metastases, the liver-first group had OS similar to the primary-first group (at 3 years: 72.2% vs. 65.6%, HR 1.164, *p =* 0.416), but longer than the simultaneous group (58.1%, HR 1.414, *p* = 0.076) (Fig. 2a). After propensity score matching, we compared (1) 84 patients in the liver-first group versus 168 patients in the primary-first group; and (2) 73 patients in the liver-first group versus 73 patients in the simultaneous group. The characteristics of the groups were similar after matching (Table 2). The liver-first and primary-first groups had similar survival (3-year OS 78.0% vs. 70.4%; *p =* 0.551), while the liver-first group had slightly longer survival than the simultaneous group (73.2% vs. 59.7%; *p* = 0.239) (Fig. 2b, c). Multivariable analyses in matched groups showed no association between treatment strategy and OS after liver resection. Similarly, there was no evidence of an association between treatment strategy and OS since CRLM diagnosis, OS after completion of treatment strategy, and RFS (data not shown).

#### Multiple Bilobar Metastases

**Fig. 3:**
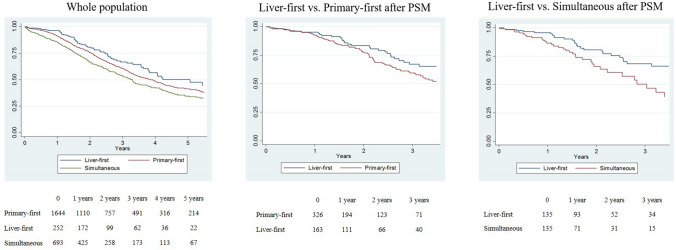
Overall survival in patients with multiple bilobar metastases according to the treatment strategy. **a** Whole population, **b** liver-first group versus primary-first group after PSM, **c** liver-first group verss simultaneous group after PSM. *PSM* propensity score matching

In patients with multiple bilobar metastases, the liver-first group had longer survival than both the primary-first group (at 3 years: 65.9% vs. 60.4%, HR 1.321, *p =* 0.031) and the simultaneous group (54.4%, HR 1.624, *p* < 0.001) (Fig. 3a). After propensity score matching, we compared (1) 163 patients in the liver-first group versus 326 patients in the primary-first group; and (2) 135 patients in the liver-first group versus 135 patients in the simultaneous group. The characteristics of the groups were similar after matching (Table 2). In both analyses, the liver-first group had longer OS after liver resection (vs. the primary-first group: 3-year OS 67.1% vs. 59.6%, *p =* 0.064; vs. the simultaneous group: 68.5% vs. 50.3%, *p* = 0.017) (Fig. 3b, c). Multivariable analyses confirmed the liver-first approach as an independent positive prognostic factor in comparison with both the primary-first approach (HR 1.667; *p =* 0.017) and the simultaneous approach (HR 2.278; *p* = 0.003) (Table 3). At multivariable analysis, the liver-first approach was associated with longer RFS than the primary-first approach (at 3 years: 37.4% vs. 26.2%, HR 1.440, *p* = 0.005). The recurrence rate was lower in the liver-first group (39.8% [64/161] vs. 49.5% [160/323]), but recurrence site was similar between the two groups (electronic supplementary Table 3). The liver-first group had borderline significantly better RFS than the simultaneous group (40.7% vs. 34.3%, HR 1.335, *p* = 0.050). At multivariable analysis, the liver-first approach was also associated with longer OS since CRLM diagnosis (vs. primary-first approach: HR 1.656, *p* = 0.020; vs. simultaneous resection: HR 2.248, *p* = 0.003), and OS after completion of the treatment strategy (vs. primary-first approach: HR 1.561, *p* = 0.040; vs. simultaneous resection: HR 1.946, *p* = 0.015).

**Table 3:** Multivariable analysis of predictive factors of overall survival in patients with multiple bilobar metastases after propensity score matching (simultaneous vs. staged groups)

Parameter	*p*-Value	HR (95% CI)
*Liver-first vs. primary-first*		
Surgical strategy		
Liver-first vs. primary-first	0.017	1.667 (1.094–2.545)
Age		
> 70 vs. ≤ 70 years	0.733	1.092 (0.657–1.817)
Year of resection		
2000–2006		1
2007–2011	0.558	0.806 (0.392–1.658)
2012–2017	0.344	0.686 (0.314–1.498)
Primary tumor site		
Left colon		1
Right/transverse colon	0.483	0.802 (0.434–1.483)
Rectum	0.532	1.146 (0.747–1.759)
N status primary tumor		
N+ vs. N0	0.283	1.266 (0.823–1.948)
Number of metastases > 3		
Y vs. N	0.900	0.975 (0.656–1.448)
Metastases size > 50 mm		
Y vs. N	0.533	0.876 (0.576–1.329)
Preoperative chemotherapy		
Y vs. N	0.594	0.861 (0.497–1.492)
Complete resection^a^		
R1 vs. R0	0.020	1.639 (1.080–2.488)
Associated intraoperative thermal ablation		
Y vs. N	0.396	1.206 (0.783––1.857)

*Liver-first *vs.* simultaneous*		
Surgical strategy		
Liver-first vs. simultaneous	0.003	2.278 (1.319–3.937)
Age		
> 70 vs. ≤ 70 years	0.703	1.137 (0.587–2.202)
Year of resection		
2000–2006		1
2007–2011	0.843	1.099 (0.431–2.800)
2012–2017	0.867	0.911 (0.305–2.720)
Primary tumor site		
Left colon		1
Right/transverse colon	0.938	0.971 (0.460–2.050)
Rectum	0.707	0.889 (0.481–1.642)
N status primary tumor		
N+ vs. N0	0.068	1.860 (0.955–3.621)
Number of metastases > 3		
Y vs. N	0.693	1.124 (0.630–2.004)
Metastases size > 50 mm		
Y vs. N	0.944	1.020 (0.585–1.780)
Preoperative chemotherapy		
Y vs. N	0.919	0.964 (0.473–1.964)
Complete resection^a^		
R1 vs. R0	0.616	0.832 (0.405–1.707)
Associated intraoperative thermal ablation		
Y vs. N	0.432	1.266 (0.703–2.281)

*HR* hazard ratio, *CI* confidence intervals, *Y* yes, *N* no

^a^R0/R1 resection refers to the surgical margin of liver resection

## Discussion

The liver-first approach was initially proposed to include radiotherapy in locally advanced rectal tumors with hepatic metastases^11^ but has generated much interest and obtained good diffusion among patients with synchronous CRLM because of its prioritization to liver disease. Some recent population-based analyses reported its application in up to 20–40% of patients.^17^,^18^,^26^ Nevertheless, the evaluation of its results still relies on a few studies collecting a limited number of cases.^19^ The snapshot from the LiverMetSurvey registry confirmed that the reverse strategy has been more and more applied since its proposal, passing from 2% before 2007 to 13% in the most recent years. This gave us the possibility of analyzing a large series including more than 550 patients. The liver-first approach was preferentially applied to patients with rectal tumors and high liver tumor burden (one-quarter of cases in the most recent period). We confirmed the expected benefits from reverse strategy: shorter chemotherapy before liver resection with an excellent response rate, and higher inclusion of pelvic radiotherapy. Nevertheless, the role of the liver-first approach in synchronous CRLM is still to be elucidated and its oncologic superiority over the other strategies is still to be proven.

Several studies focused on the short-term results of the three approaches, most comparing simultaneous and staged procedures with controversial results.^27^–^32^ The comparison between the primary-first and liver-first approaches did not generate much interest because, as expected, the two had similar outcomes through all studies.^12^,^13^,^15^–^17^,^21^ In the present series, the three approaches had equivalent results for low-complexity resections, while the simultaneous group had worse outcomes than staged procedures for major hepatectomies and resections of multiple bilobar metastases. Our results are coherent with those recently published by Shubert et al. based on a large US database (*n* = 43,408).^30^ They demonstrated that the operative risks of simultaneous resections vary incrementally with the complexity of both hepatectomy and colorectal resection. Even if our picture is incomplete because we lack data about the morbidity of primary tumor resection in staged procedures, we believe that the increased mortality risk of the simultaneous approach in complex procedures is a major point.

Data regarding the long-term outcome are scarce. In 2012, the Geneva group analyzed the LiverMetSurvey registry and demonstrated non-inferiority, but not superiority, of the liver-first approach versus the primary-first approach.^15^ Other papers confirmed these data,^12^,^13^,^15^–^22^,^33^,^34^ and, to date, no studies have reported a survival advantage of the liver-first approach over the other approaches. Coherently, the EGOSLIM (Expert Group on OncoSurgery management of LIver Metastases) group,^6^ stated that simultaneous resection, when feasible without increasing operative risk, is the preferred option, while one of the two-staged procedures (primary-first or liver-first) should be pursued for the remaining patients. A recent network meta-analysis confirmed that no strategy to resect synchronous CRLM has superiority over the others in terms of survival, even if the liver-first approach was ranked as the best treatment for its relative efficacy based on 5-year OS outcomes.^19^ This evidence is too weak to propose the reverse strategy as standard.

All studies that compared strategies for synchronous CRLM suffered from major heterogeneity among groups. Different approaches are scheduled for different patients. In the literature,^17^–^19^ as in the present series, patients undergoing a reverse approach have more advanced liver disease and more rectal tumors. To face this scenario, we decided to not only adopt a propensity score match to make populations comparable but to also stratify patients according to their hepatic tumor burden. We hypothesized that the surgical strategy may have a different impact on prognosis according to the severity of the disease.

Our hypothesis was confirmed. The liver-first approach had results similar to the other approaches in patients with unilobar synchronous CRLM but was associated with a clear survival advantage over both the primary-first and simultaneous approaches in patients with multiple bilobar metastases. Its superiority was confirmed on multivariable analysis on the whole series and after strict propensity score matching. Three-year survival rates after reverse strategy exceeded 65% and ranged between 50 and 60% in the other groups. These results are even more relevant if we consider that the primary-first group included only patients who completed the two resections, but not those who dropped out. Intention-to-treat analysis is expected to show an even wider difference in favor of the liver-first approach. Factors contributing to these results are still to be investigated, but some hypotheses can be advanced. First, reverse strategy prioritizes the treatment of the tumor site that is judged the most prognostically relevant, i.e. the liver. Second, the liver-first approach requires early management of patients by an expert multidisciplinary liver team. Some studies demonstrated that such management is associated with longer survival.^35^–^37^ Third, the reverse strategy maximizes the effectiveness of neoadjuvant chemotherapy because liver surgery is performed with optimal timing, without any treatment interruption, and at the peak of the response. Any delay in surgery after chemotherapy may lead to early tumor reactivation and poor prognosis.^38^,^39^ Fourth, in the primary-first approach, colorectal resection is associated with an immunological alteration that could favor liver metastases proliferation.^40^ Finally, in comparison with simultaneous resections, the liver-first approach minimized postoperative morbidity that may negatively impact prognosis.^41^

**Fig. 4:**
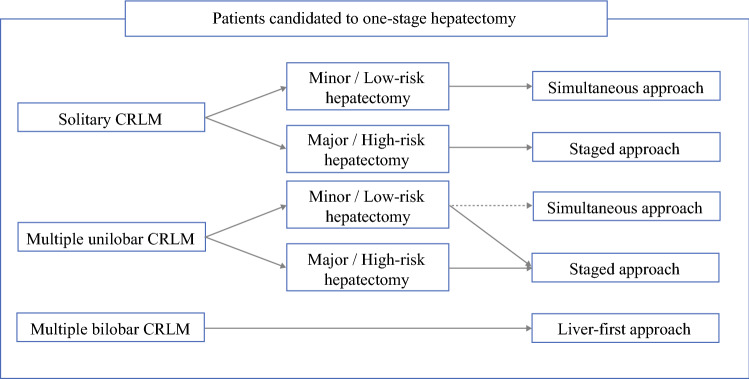
Treatment strategy of synchronous colorectal liver metastases according to hepatic tumor burden and scheduled hepatectomy. *CRLM* colorectal liver metastases

The main strength of this study was its clinical relevance. We proposed a tumor burden-driven strategy for patients with synchronous CRLM as candidates for a single-stage hepatectomy (Fig. 4). According to our results, the liver-first approach should be the preferred option for patients with multiple bilobar metastases, while simultaneous resection should be the preferred option for patients with solitary metastasis requiring a minor hepatectomy, even if the definition of ‘minor hepatectomies’ is debated.^42^ In the remaining patients, the staged procedures were equivalent, while simultaneous resections should be cautiously considered because they have increased mortality risk whenever a major hepatectomy is needed. We did not analyze patients undergoing a two-stage hepatectomy, but, according to the favorable results that we observed in patients with simultaneous colorectal resection and unilobar limited resections, we can assume that the first stage of a two-stage hepatectomy can be safely combined with colorectal resection. Some limitations could be argued. This was a retrospective non-intention-to-treat analysis and the three groups had major heterogeneity at baseline. However, we collected a large number of patients from several centers worldwide and performed an accurate propensity score matching to make groups comparable, even if it led to a major reduction in sample size. Some prognostic data, such as CEA or RAS mutational status, were not considered and should be the subject of further analyses.

## Conclusion

The surgical strategy in patients with colorectal cancer and synchronous liver metastases should be decided according to the hepatic tumor burden. In patients with multiple bilobar CRLM, we strongly suggest the liver-first approach as the standard because it is associated with excellent short-term results and longer survival than the alternative approaches.

## Disclosure

Santiago López-Ben has received grants from Olympus, Baxter and Integra, and Rene Adam has received honoraria for participation at meetings or congresses organized by Sanofi or Merck Serono. Felice Giuliante, Luca Viganò, Agostino M. De Rose, Darius F. Mirza, Réal Lapointe, Gernot Kaiser, Eduardo Barroso, Alessandro Ferrero, Helena Isoniemi, Irinel Popescu, Jean-Francois Ouellet, Catherine Hubert, Jean-Marc Regimbeau, Jen-Kou Lin, Oleg G. Skipenko, and Francesco Ardito have no conflicts of interest to declare.

## Supplementary Information

Below is the link to the electronic supplementary material.

Supplementary file1 (JPG 140 kb)

Supplementary file2 (DOCX 34 kb)

Supplementary file3 (DOCX 24 kb)
