# Supplementary figures and images for: Liver-First Approach for Synchronous Colorectal Metastases: Analysis of 7360 Patients from the LiverMetSurvey Registry
Source: Ann Surg Oncol. 2021 Jul 1;28(13):8198–208. doi: 10.1245/s10434-021-10220-w (PMC8590998; doi:10.1245/s10434-021-10220-w)

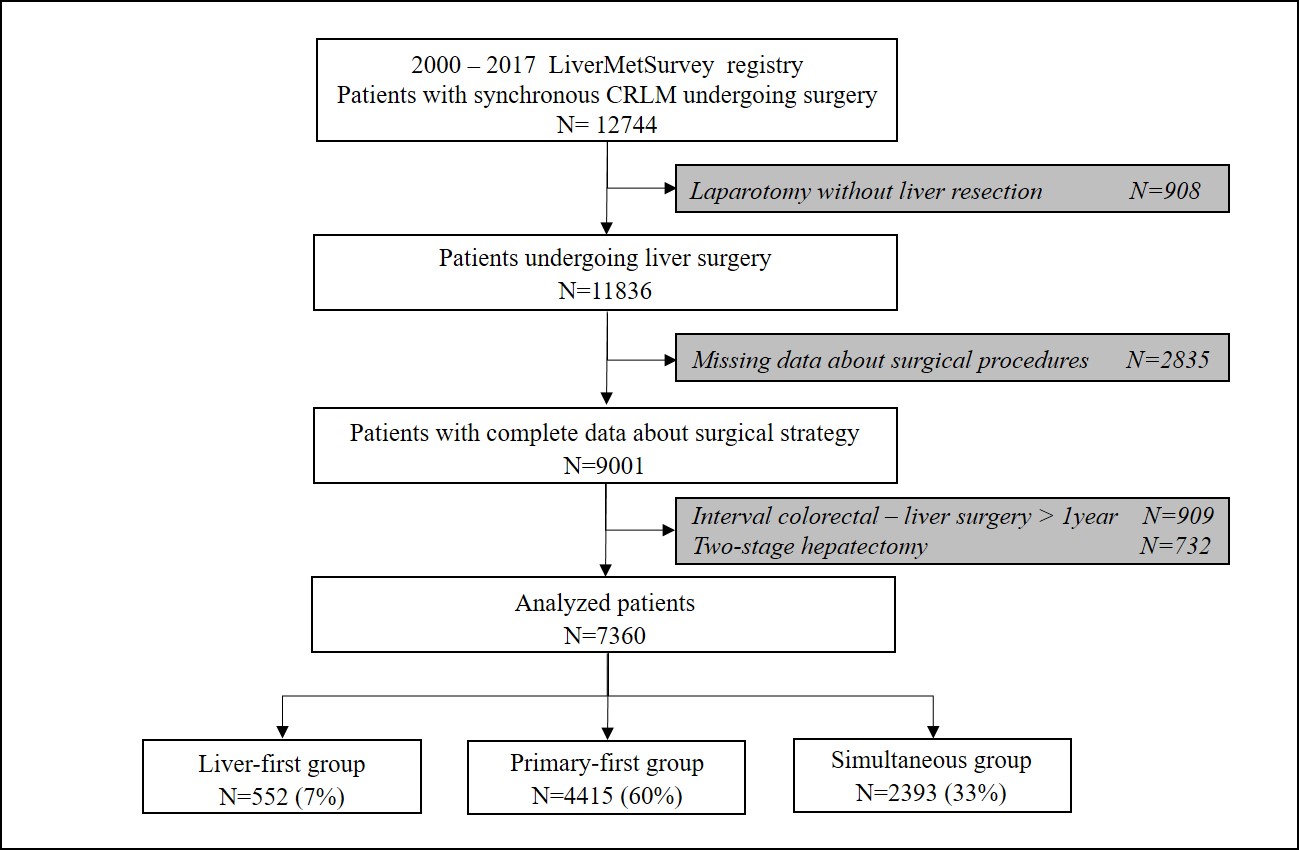

Supplement: Supplementary file 1 — Supplementary file1 (JPG 140 kb) [file 10434_2021_10220_MOESM1_ESM.jpg]
