# Supplementary material for: Liver-First Approach for Synchronous Colorectal Metastases: Analysis of 7360 Patients from the LiverMetSurvey Registry
Source: Ann Surg Oncol. 2021 Jul 1;28(13):8198–208. doi: 10.1245/s10434-021-10220-w (PMC8590998; doi:10.1245/s10434-021-10220-w)
Supplement: Supplementary file 2 — Supplementary file2 (DOCX 34 kb) [file 10434_2021_10220_MOESM2_ESM.docx]

**Supplementary Figure 2.** Overall survival according to the treatment strategy in the whole series


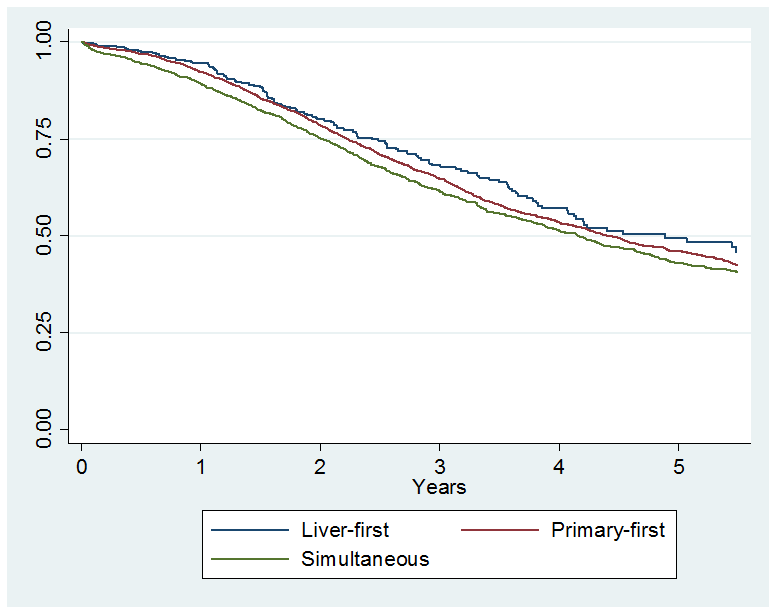


|  | 0 | 1 year | 2 years | 3 years | 4 years | 5 years |
| --- | --- | --- | --- | --- | --- | --- |
| Primary-first | 4415 | 3052 | 2163 | 1461 | 995 | 688 |
| Liver-first | 552 | 359 | 216 | 139 | 82 | 49 |
| Simultaneous | 2393 | 1527 | 1032 | 678 | 441 | 289 |
