# Supplementary material for: Liver-First Approach for Synchronous Colorectal Metastases: Analysis of 7360 Patients from the LiverMetSurvey Registry
Source: Ann Surg Oncol. 2021 Jul 1;28(13):8198–208. doi: 10.1245/s10434-021-10220-w (PMC8590998; doi:10.1245/s10434-021-10220-w)
Supplement: Supplementary file 3 — Supplementary file3 (DOCX 24 kb) [file 10434_2021_10220_MOESM3_ESM.docx]

**Supplementary Table 1**. Short-term results according to the surgical strategy

| **Variable**  **# / available (%)** | **Liver-first** | **Primary-first group** | **P**  **Liver-first vs. Primary-first** | **Simultaneous group** | **P**  **Liver-first vs. Simultaneous** | **P**  **Staged vs. Simultaneous** |
| --- | --- | --- | --- | --- | --- | --- |
| **90-day mortality** | | | | | | |
| **Whole series** | 14/548 (2.6%) | 74/4353 (1.7%) | 0.156 | 74/2337 (3.2%) | 0.454 | <0.001 |
| **If major hepatectomy** | 7/145 (4.8%) | 35/1202 (2.9%) | 0.210 | 23/285 (8.1%) | 0.212 | <0.001 |
| **Solitary metastasis** | 3/143 (2.1%) | 20/1482 (1.4%) | 0.469 | 23/1092 (2.1%) | 0.995 | 0.171 |
| **Multiple unilobar metastasis** | 5/134 (3.7%) | 19/1067 (1.8%) | 0.128 | 13/493 (2.6%) | 0.501 | 0.414 |
| **Multiple bilobar metastasis** | 6/265 (2.3%) | 35/1748 (2.0%) | 0.779 | 37/724 (5.1%) | 0.052 | <0.001 |
| **Overall morbidity** | | | | | | |
| **Whole series** | 150/494 (30.4%) | 1151/4073 (28.3%) | 0.328 | 713/2074 (34.4%) | 0.090 | <0.001 |
| **If major hepatectomy** | 49/133 (36.8%) | 392/1134 (34.6%) | 0.602 | 121/264 (45.8%) | 0.087 | 0.001 |
| **Solitary metastasis** | 35/130 (26.9%) | 356/1399 (25.5%) | 0.712 | 305/972 (31.4%) | 0.302 | 0.002 |
| **Multiple unilobar metastasis** | 37/119 (31.1%) | 288/995 (28.9%) | 0.626 | 146/443 (33.0%) | 0.700 | 0.143 |
| **Multiple bilobar metastasis** | 75/241 (31.1%) | 499/1640 (30.4%) | 0.827 | 258/646 (39.9%) | 0.016 | <0.001 |
| **Hepatic complications** | | | | | | |
| **Whole series** | 73/492 (14.8%) | 621/4066 (15.3%) | 0.799 | 292/2057 (14.2%) | 0.715 | 0.276 |
| **If major hepatectomy** | 27/133 (20.3%) | 227/1132 (20.1%) | 0.946 | 60/258 (23.3%) | 0.506 | 0.272 |
| **Solitary metastasis** | 14/130 (10.8%) | 166/1398 (11.9%) | 0.709 | 110/967 (11.4%) | 0.838 | 0.759 |
| **Multiple unilobar metastasis** | 18/117 (15.4%) | 157/946 (16.6%) | 0.739 | 60/424 (14.2%) | 0.737 | 0.270 |
| **Multiple bilobar metastasis** | 32/226 (14.2%) | 263/1516 (17.4%) | 0.233 | 114/597 (19.1%) | 0.098 | 0.230 |
| **Infectious complications** | | | | | | |
| **Whole series** | 58/492 (11.8%) | 462/4066 (11.4%) | 0.779 | 383/2058 (18.6%) | <0.001 | <0.001 |
| **If major hepatectomy** | 20/133 (15.0%) | 142/1132 (12.5%) | 0.416 | 55/258 (21.3%) | 0.135 | <0.001 |
| **Solitary metastasis** | 16/130 (12.3%) | 160/1398 (11.4%) | 0.768 | 174/967 (18.0%) | 0.108 | <0.001 |
| **Multiple unilobar metastasis** | 13/117 (11.1%) | 96/946 (10.1%) | 0.746 | 81/425 (19.1%) | 0.044 | <0.001 |
| **Multiple bilobar metastasis** | 27/226 (12.0%) | 179/1516 (11.8%) | 0.952 | 120/597 (20.1%) | 0.006 | <0.001 |

**Supplementary Table 2.** Multivariable analysis of predictive factors of overall survival

| **Parameter** | | **Whole series** | |
| --- | --- | --- | --- |
|  |  | **P** | **HR (CI95%)** |
| Age | >70 vs. ≤70 years | <0.001 | 1.470 (1.311-1.649) |
| Year of resection | 2000-2006 |  | 1 |
|  | 2007-2011 | 0.774 | 0.984 (0.878-1.101) |
|  | 2012-2017 | 0.024 | 0.826 (0.700-0.975) |
| Surgical strategy | Liver-first |  | 1 |
|  | Primary-first | 0.100 | 1.212 (0.964-1.524) |
|  | Simultaneous | 0.036 | 1.298 (1.017-1.656) |
| Primary tumor site | Left colon |  | 1 |
|  | Right/Transverse colon | <0.001 | 1.379 (1.219-1.561) |
|  | Rectum | <0.001 | 1.256 (1.115-1.415) |
| N status primary tumor | N+ vs. N0 | <0.001 | 1.632 (1.450-1.837) |
| Number of metastases >3 | Y vs. N | <0.001 | 1.295 (1.134-1.481) |
| Metastasis size >50 mm | Y vs. N | <0.001 | 1.246 (1.110-1.397) |
| Bilobar metastases | Y vs. N | 0.021 | 1.153 (1.021-1.301) |
| Extrahepatic disease | Y vs. N | 0.221 | 1.224 (0.886-1.691) |
| Preoperative chemotherapy | Y vs. N | 0.034 | 0.890 (0.799-0.991) |
| Complete resection * | R1 vs. R0 | 0.088 | 1.129 (0.982-1.298) |
| Associated i.o. thermal ablation | Y vs. N | 0.002 | 1.285 (1.097-1.506) |
| *HR: hazard ratio; CI: confidence intervals; Y: yes; N: No; TBS: tumor burden score; Y: yes; N: no; i.o.: intraoperative; preop.: preoperative*  ** R0/R1 resection refers to the surgical margin of liver resection* | | | |

**Supplementary Table 3.** Recurrence site in patients with multiple bilobar metastases

| Recurrence site | Strategy | | P |
| --- | --- | --- | --- |
|  | Liver-first group | Colon-first group |  |
| Hepatic | 38 (57.6%) | 80 (50.0%) | 0.269 |
| Extrahepatic | 24 (36.4%) | 59 (36.9%) |  |
| Hepatic + extrahepatic | 4 (6.1%) | 21 (13.1%) |  |
